# Clinical insights gained by refining the 2016 WHO classification of diffuse gliomas with: *EGFR* amplification, *TERT* mutations, *PTEN* deletion and *MGMT* methylation

**DOI:** 10.1186/s12885-019-6177-0

**Published:** 2019-10-17

**Authors:** Cheila Brito, Ana Azevedo, Susana Esteves, Ana Rita Marques, Carmo Martins, Ilda Costa, Manuela Mafra, José M. Bravo Marques, Lúcia Roque, Marta Pojo

**Affiliations:** 1 Unidade de Investigação em Patobiologia Molecular (UIPM) do Instituto Português de Oncologia de Lisboa Francisco Gentil E.P.E., Rua Prof. Lima Basto, 1099-023 Lisbon, Portugal; 2 Serviço de Neurologia do Instituto Português de Oncologia de Lisboa Francisco Gentil E.P.E., Rua Prof. Lima Basto, 1099-023 Lisbon, Portugal; 3 Faculty of Health Sciences, University of Beira Interior, 6200-506 Covilhã, Portugal; 4 Unidade de Investigação Clínica (UIC) do Instituto Português de Oncologia de Lisboa Francisco Gentil E.P.E., Rua Prof. Lima Basto, 1099-023 Porto, Portugal; 5 Serviço de Anatomia Patológica do Instituto Português de Oncologia de Lisboa Francisco Gentil E.P.E, Rua Prof. Lima Basto, 1099-023 Lisbon, Portugal

**Keywords:** *EGFR*, *TERT*, *MGMT*, *PTEN*, *IDH*, 1p/19q codeletion, 2016 WHO classification, Gliomas

## Abstract

**Background:**

Significant advances in the molecular profiling of gliomas, led the 2016 World Health Organization (WHO) Classification to include, for the first-time, molecular biomarkers in glioma diagnosis: *IDH* mutations and 1p/19q codeletion. Here, we evaluated the effect of this new classification in the stratification of gliomas previously diagnosed according to 2007 WHO classification. Then, we also analyzed the impact of *TERT* promoter mutations, *PTEN* deletion, *EGFR* amplification and *MGMT* promoter methylation in diagnosis, prognosis and response to therapy in glioma molecular subgroup.

**Methods:**

A cohort of 444 adult gliomas was analyzed and reclassified according to the 2016 WHO. Mutational analysis of *IDH1* and *TERT* promoter mutations was performed by Sanger sequencing. Statistical analysis was done using SPSS Statistics 21.0.

**Results:**

The reclassification of this cohort using 2016 WHO criteria led to a decrease of the number of oligodendrogliomas (from 82 to 49) and an increase of astrocytomas (from 49 to 98), while glioblastomas (GBM) remained the same (*n* = 256). GBM was the most common diagnosis (57.7%), of which 55.2% were *IDH-*wildtype. 1p/19q codeleted gliomas were the subgroup associated with longer median overall survival (198 months), while GBM *IDH-*wildtype had the worst outcome (10 months). Interestingly, *PTEN* deletion had poor prognostic value in astrocytomas *IDH-*wildtype (*p* = 0.015), while in GBM *IDH-*wildtype was associated with better overall survival (*p* = 0.042) as well as *MGMT* promoter methylation (*p* = 0.009). *EGFR* amplification and *TERT* mutations had no impact in prognosis. Notably, *EGFR* amplification predicted a better response to radiotherapy (*p* = 0.011) and *MGMT* methylation to chemo-radiotherapy (*p* = 0.003).

**Conclusion:**

In this study we observed that the 2016 WHO classification improved the accuracy of diagnosis and prognosis of diffuse gliomas, although the available biomarkers are not enough. Therefore, we suggest *MGMT* promoter methylation should be added to glioma classification. Moreover, we found two genetic/clinical correlations that must be evaluated to understand their impact in the clinical setting: i) how is *PTEN* deletion a favorable prognostic factor in GBM *IDH* wildtype and an unfavorable prognostic factor in astrocytoma *IDH* wildtype and ii) how *EGFR* amplification is an independent and strong factor of response to radiotherapy.

## Background

Diffuse gliomas are one of the most common primary neoplasms of the central nervous system, accounting for approximately 81% of all malignant brain tumors, leading to a high rate of mortality and morbidity [1, 2]. These aggressive and heterogeneous tumors are generally associated with poor outcomes, due to their complexity and resistance to therapeutic approaches [3].

In the last years, improvements in molecular techniques have been important tools to update the knowledge about the genetic profile of gliomas. These progresses, led in 2016, the World Health Organization (WHO) classification of Central Nervous System Tumors to include Isocitrate dehydrogenase (*IDH)* mutations and 1p/19q codeletion as central biomarkers for the diagnosis of diffuse gliomas [4]. This new classification breaks the principle of diagnosis based exclusively on microscopy, allowing a more accurate determination of the patient’s prognosis [4, 5]. Nevertheless, this new classification has limitations to characterize these heterogeneous tumors. New biomarkers for diagnostic, prognostic and response to therapy are a major concern for the management of patients with gliomas [6]. In this context, different potential biomarkers for diffuse gliomas have been proposed, such as: *TERT* (telomerase reverse transcriptase) promoter mutations, amplification/mutations in *EGFR* (epidermal growth factor receptor) gene, mutations/deletions in *PTEN* (phosphatase and tensin homologue) and *MGMT* (O-6-methylguanine-DNA methyltransferase) promoter methylation.

*TERT* promoter mutations are present in a high percentage of gliomas (80–90%), which makes it an interesting target gene to be studied [7]. This gene encodes the catalytic subunit of telomerase, an enzyme that maintains the length of telomeres during cell division [8]. In addition, *TERT* promoter mutations are associated with increased levels of telomerase activity allowing the indefinite proliferation of tumor cells [8–10]. The amplification of *EGFR* was identified in approximately 40–50% of all cases of glioblastoma (GBM), 2007 WHO grade IV, the most malignant of diffuse gliomas [11, 12]. This molecular alteration determines the over-activation of an important signaling pathway, phosphatidylinositol-3-kinase - protein kinase B (PI3K-AKT), which regulates a wide range of cellular processes such as cell proliferation, migration, angiogenesis, differentiation and apoptosis [13].

*PTEN* deletion is present in approximately 30–40% of GBM [14, 15], however there is no unanimity regarding the prognostic value of this alteration in diffuse gliomas [16, 17], as well as, regarding *TERT* promoter mutations [18–20] and *EGFR* amplification [21–23].

*MGMT* promoter methylation has been described as a predictive biomarker in GBM with benefit from chemotherapy based on temozolomide [24–26]. Moreover, this benefit is higher in patients with *IDH*-wildtype gliomas, particularly in old patients (aged ≥70 years) [3, 27, 28]. *MGMT* promoter methylation is predominant in *IDH*-mutant gliomas, representing a favorable prognostic factor, although this biomarker is not associated with the benefit from either temozolomide or radiotherapy in this molecular subgroup [27].

Currently, these genes are not included in the 2016 WHO classification of diffuse gliomas, although these genetic alterations could be relevant in the diagnostic routine, patient management and on the choice of the treatments [4, 29].

In the present study, we aimed to reclassify a 444 cohort of diffused gliomas based on the 2016 WHO classification of Central Nervous System Tumors. Subsequently, we used this reclassified cohort to evaluate the impact of *TERT* promoter mutations, *PTEN* deletion, *EGFR* amplification and *MGMT* promoter methylation in diagnosis, prognosis and response to therapy.

## Material and methods

### Biological samples

A dataset of adult diffuse glioma samples was obtained from patients diagnosed from 2011 to 2016, in Unidade de Investigação em Patobiologia Molecular of Instituto Português de Oncologia de Lisboa Francisco Gentil (IPOLFG). In this study we included all consecutive glioma patients referred for treatment in our center, previously submitted to surgery, with known histologic diagnosis and biological material available. This study was previously approved by the IPOLFG Ethical Board Committee. Four hundred forty-four glioma samples were reclassified according to the 2016 WHO classification. However, statistical analysis was performed using only 403 samples, due to the exclusion of the NOS (Not Otherwise Specified) glioma group. These samples were previously characterized in the diagnostic routine for: *IDH* mutations and *TERT* promoter mutations by Sanger Sequencing. *MGMT* promoter methylation was determined by Multiplex Ligation-dependent Probe Amplification (MLPA – MRC-Holland according with guidelines defined by van den Bent [30]). *PTEN* deletion (Vysis PTEN/CEP10), *EGFR* amplification (Vysis EGFR/CEP7) and 1p/19q codeletion (Vysis,1p36/1q25 and 19q13/19.13 dual color probe) were identified by Fluorescent in situ hybridization (FISH). The definition of numerical alterations was performed according the FISH criteria defined by the International System of Human Cytogenetic Nomenclature (ISCN) 2016 [31].

### DNA extraction

For samples without the mutational status of *IDH1* (*n* = 92) and *TERT* promoter mutations (*n* = 82), we extracted DNA, when not available from the routine diagnosis, which was used at a concentration of 80 ng/μl. Tumor samples were received as fresh tissue or paraffin-preserved tissue for DNA extraction. The tumor sections for DNA extraction were selected by a neuropathologist consider the following criteria: 1) cellular regions without necrosis, 2) representative regions of tumor subtype and 3) areas with a minimum of 2 mm of diameter. The DNA extraction from frozen tissues was performed using the conventional method of phenol-chloroform (MERCK, Germany). From tissues fixed in formaldehyde and preserved in paraffin, the DNA was isolated using the QIAGEN’s Gene Read™ DNA FFPE Kit. Additionally, for some samples included in this project the DNA was extracted using an automatized process by Maxwell® RSC Instrument (Promega, USA), using the RSC DNA FFPE kit (Promega, USA). The extraction was performed according to the manufacture’s protocol. The DNA concentration and quality were assessed using Nanodrop 2000 (Thermo Fisher Scientific, USA).

### Polymerase chain reaction (PCR) and sequencing

The mutational analysis directed to exon 4 of *IDH1* and *TERT* promoter was performed using two sets of primers for the detection of hotspot mutations: missense mutations involving a single amino acid change at arginine 132 (R132) of *IDH1* and C228T and C250T map − 124 and − 146 bp upstream of *TERT* ATG site. The target amplification of *IDH1* was achieved using the forward primer 5′ CGGTCTTCAGAGAAGCCATT 3′ and the reverse primer 5′ GCAAAATCACATTATTGCCAAC3’ and *TERT* promoter was amplified using the forward primer 5′ GCACAGACGCCCAGGACCGCGCT 3′ and the reverse primer 5′ TTCCCACGTGCGCAGCAGGACGCA 3′ generating fragments with 129 bp and 196 bp respectively. PCR contained 35 cycles with annealing at 56 °C for *IDH1* and 69.5 °C for *TERT* promoter. Then, an enzymatic method was used to purify each PCR product, using two distinct enzymes: Exonuclease I 20 U/μl (Thermo Fisher Scientific, USA) and FastAP Thermosensitive Alkaline Phosphatase 1 U/μl (Thermo Fisher Scientific, USA). To determine the sequence of interest in *IDH1* and *TERT* promoter gene, an automatic sequencer was used, ABI PrismTM 3130 Genetic Analyser (Applied Biosystems, USA) following the protocol purposed by Big Dye™ Terminator v1.1 Cycle Sequencing Kit (Applied Biosystems, USA).

### Statistical analysis

The primary endpoint was overall survival, defined as the time from the glioma diagnosis to the patient death or last follow up. Survival analysis was done using Kaplan-Meier estimator and the log-rank test for group comparison. Variables with a significant *p-value* in the univariate analysis were exposed to a multivariate analysis using Cox regression proportional hazard model. The multivariate analysis allowed to study the independent association of the molecular subgroups established with overall survival, while controlling for potential confounders such as age, sex and treatment. In order to eliminate confounder variables, the number of cases of each subtype was reduced because the type of treatment was not accessible for all the cases included in the cohort. Additionally, to evaluate the association between the interest biomarkers and the overall survival was performed a multivariate analysis controlling for: *MGMT* methylation, *PTEN* deletion and *EGFR* amplification*. TERT* promoter mutations were excluded from this analysis, since the number of samples would reduce the dataset available to determine the impact of the remaining biomarkers.

All tests were two-sided, and we considered a significance level of 5%. The statistical analysis applied here was performed using IBM SPSS Statistics 21.0.

## Results

### The impact of 2016 WHO classification in the stratification of diffuse gliomas

The reorganization of diffuse gliomas according to 2016 WHO classification mainly affected oligodendroglioma and astrocytoma subgroups, reducing the number of oligodendrogliomas (82 to 49) and increasing the astrocytomas (49 to 98), while the number of GBM remained the same (Table 1). Additionally, 41 samples were not included in any glioma subset (Glioma NOS), mainly due to: technical issues, samples with only 1p or 19q deletion or with 1p/19q codeletion and *IDH*-wildtype. Importantly, in this new classification the subgroup of oligoastrocytomas was reorganized between astrocytomas, oligodendrogliomas or NOS.

**Table 1:** Effect of 2016 WHO classification on the subdivision of glioma subgroups

		WHO Classification 2016				Totals
		GBM (*IDH* wt/*IDH* mut)	1p/19q codeleted and *IDH* mut	Astrocytoma *(IDH* wt/*IDH* mut)	NOS	
WHO Classification 2007	GBM	256	0	0	0	256
	Oligodendrogliomas (Grade II/III)	0	39	13	30	82 (52/30)
	Astrocytoma (Grade II/III)	0	2	46	1	49 (21/28)
	Oligoastrocytomas	0	8	35	10	53
	NOS	0	0	4	0	4
	Totals	256	49	98	41	444 (100%)

This molecular reclassification allowed the division of diffuse gliomas into 6 molecular subgroups according to *IDH* mutations and 1p/19q codeletion analysis (Table 2). GBM samples corresponded to 57.7% of the entire cohort, of which 55.2% were GBM *IDH-*wildtype and the remaining 2.5% were GBM *IDH*-mutant. The astrocytoma subgroup was the second most frequent (22.1%) and the NOS glioma subgroup corresponded to 9.2% of the cohort analyzed (Table 2). *IDH*-mutant gliomas, whether GBMs or astrocytomas, are predominant in young patients (44 and 38 years respectively) in comparison with *IDH*-wildtype gliomas (63 and 57 years respectively) (Table 3). In addition, our results also indicated a higher prevalence of GBM *IDH*-wildtype, astrocytomas *IDH*-wildtype and *IDH*-mutant and 1p/19 codeleted gliomas in men (2:1; 1.3:1; 1.4:1 and 2.1 respectively), except for the GBM *IDH-*mutant subgroup (0.8:1) (Table 3).

**Table 2:** Relative frequency of glioma molecular subgroups according to the 2016 WHO classification

Molecular Subgroups	Number of samples	% Gliomas
GBM*, IDH* mutant	11	2.5 (11/444)
GBM, *IDH-*wildtype	245	55.2 (245/444)
Astrocytoma, *IDH-*mutant	55	12.4 (55/444)
Astrocytoma, *IDH-*wildtype	43	9.7 (43/444)
*IDH* mutant and 1p/19q codeleted	49	11.0 (49/444)
NOS	41	9.2 (41/444)
Total	444	100 (444/444)

**Table 3:** Clinicopathological data of gliomas patients based on the molecular subgroups

Variable	No	Glioma Molecular subgroups				
		*GBM, IDH* wildtype	*GBM, IDH* mutant	1p/19q codeleted gliomas	*Astrocytoma, IDH* wildtype	*Astrocytoma, IDH* mutant
Number of samples	403	245	11	49	43	55
Age of diagnosis (years)	Median	63.0	44.0	48.5	57.0	38.0
	Minimum	18.0	17.0	27.0	16.0	23.0
	Maximum	87.0	59.0	76.0	80.0	66.0
Sex	Male	163	5	34	24	32
	Female	82	6	15	19	23
	Ratio (M/F)	2:1	0.8:1	2:1	1.3:1	1.4:1
Adjuvant Therapy	Receveid	211	11	30	27	45
	None	19	0	1	6	3
	No data	15	0	18	10	7

**Fig. 1:**
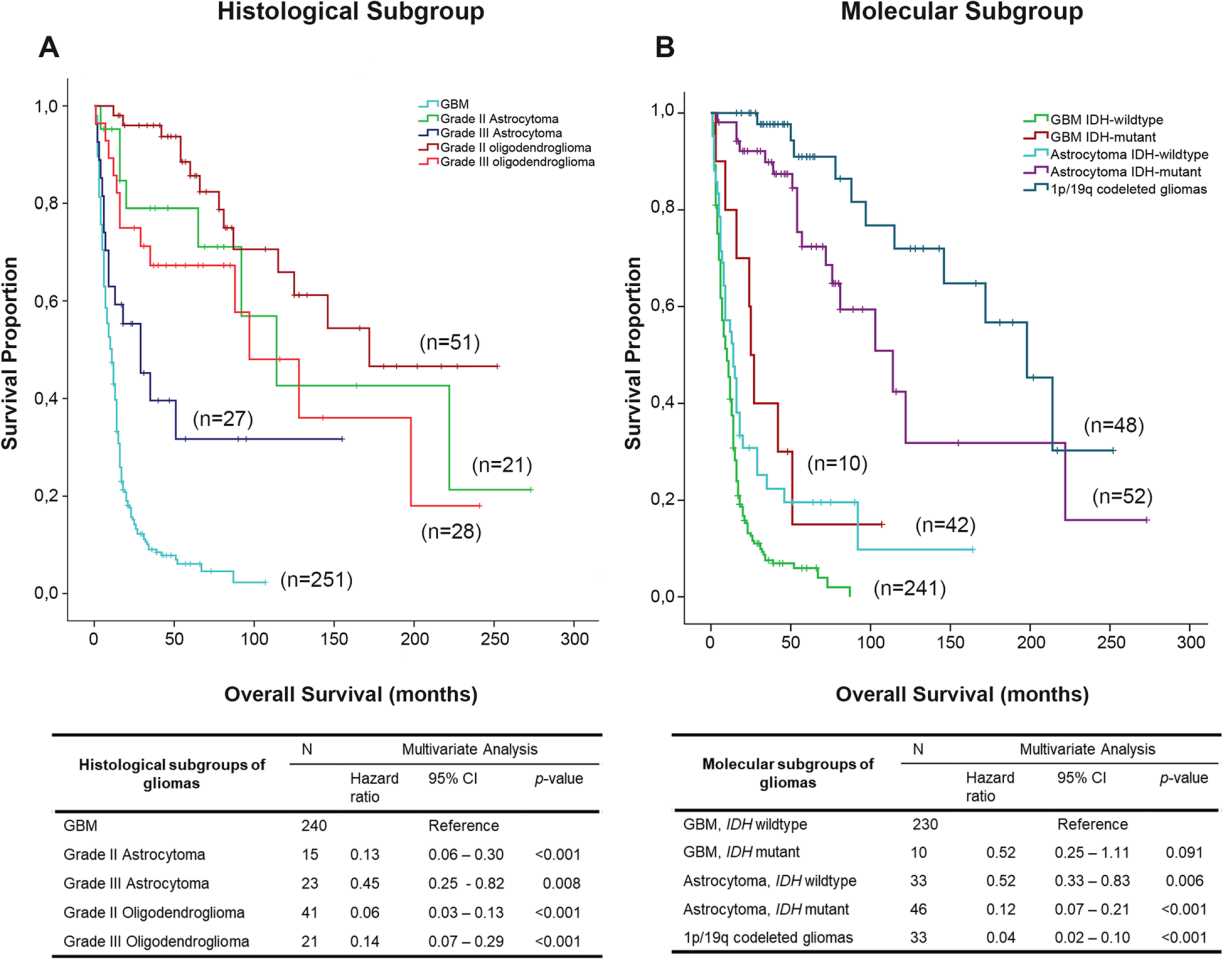
Kaplan-Meier curves of overall survival for the subgroups of gliomas established according to the 2007 (**a**) and 2016 (**b**) WHO classifications. **a** the overall survival curve of histological group (top) and the respective multivariant analysis (bottom). **b** The overall survival for the molecular subgroups of gliomas (top) and the respective multivariant analysis (bottom)

Then, we evaluated the prognostic value of histological grade and molecular subgroups. Grade II oligodendroglioma was the histological subgroup associated with longer overall survival (Median Overall Survival (OS):172 months); in contrast, GBM (grade IV) was the subgroup associated with the poorer outcome (OS:11 months) (Fig. 1). Using the molecular classification, gliomas with 1p/19q codeletion were the subgroup associated with better prognosis (OS:198 months). For the remaining molecular subgroups (GBM and astrocytomas), *IDH-*mutant tumors were associated with better prognosis (OS:25 and 114 months, respectively) when compared to the *IDH-*wildtype subgroups (OS:10 and 14 months, respectively) (Fig. 1).

The multivariate analysis performed using Cox Regression Hazard model evidenced the prognostic impact of the molecular subgroups and histological grades, after adjustment for age, gender and treatment (Fig. 1). The GBM *IDH-*mutant subgroup was the only group that did not show a statistically significant *p*-value (*p*-value = 0.092), perhaps due to the reduced number of samples (*n* = 10). These results validated the size and representativeness of each glioma molecular subgroup to perform further studies, as well as, the accuracy introduced by molecular markers in the prognosis and diagnosis of patients with gliomas.

**Table 4:** Multivariate analysis for the prognostic impact of *IDH* mutations, *PTEN* deletion, *MGMT* methylation and *EGFR* amplification

	N	Median Survival (MS-months)	N	Multivariate analysis^a^		
				Hazard ratio	95% Cl	*p*-Value
GBM						
*IDH*						
Mutant	10	25.0	10	0.52	0.24–1.11	0.092
Wildtype	241	10.0	230		Reference	
Astrocytoma						
*IDH*						
Mutant	42	14.0	33	0.22	0.11–0.43	< 0.001
Wildtype	52	114.0	46		Reference	
GBM *IDH*-wildtype						
*PTEN*						
Deleted	184	11.0	171	0.65	0.43–0.99	0.042
Non-Deleted	38	6.0	34		Reference	
*MGMT*						
Methylated	52	12.0	46	0.61	0.42–0.88	0.009
Unmethylated	180	9.0	159		Reference	
*EGFR*						
Amplified	88	11.0	82	0.88	0.65–1.18	0.393
Non- Amplified	135	8.0	123		Reference	
Astrocytoma *IDH*-wildtype						
*PTEN*						
Deleted	18	8.0	14	4.48	1.34–14.94	0.015
Non-deleted	17	29.0	11		Reference	
*MGMT*						
Methylated	9	3.0	7	0.69	0.20–2.4	0.555
Unmethylated	31	16.0	18		Reference	
*EGFR*						
Amplified	14	14.0	12	0.71	0.25–2.03	0.522
Non- Amplified	20	13.0	13		Reference	

^a^Multivariate analysis was performed controlling the following independent variables: age, gender, treatment

### The frequency of *TERT* promoter mutations, *EGFR* amplification, *PTEN* deletion and *MGMT* promoter methylation in molecular glioma subgroups

**Fig. 2:**
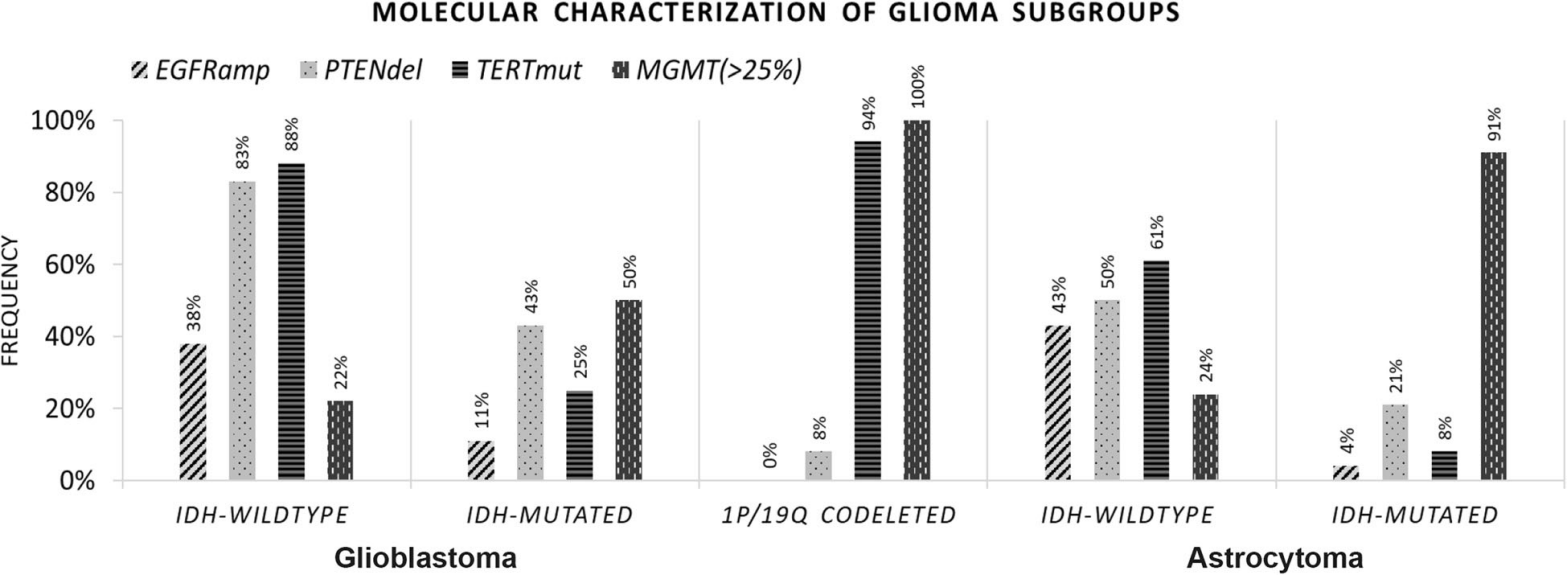
Frequency of *EGFR* amplification (amp), *PTEN* deletion (del), *TERT* promoter mutations (mut) and *MGMT* promoter methylated samples in the distinct glioma molecular subgroups. *EGFR* amplification was analyzed: in 227 GBM-*IDH* wildtype, 9 GBM *IDH*-mutant, 48 1p/19q codeleted gliomas, 35 Astrocytoma *IDH*-wildtype, 53 Astrocytomas *IDH*-mutant. *PTEN* deletion was analyzed: in 225 GBM *IDH*-wildtype, 7 GBM *IDH*-mutant, 48 1p/19q codeleted gliomas, 37 Astrocytoma *IDH*-wildtype and 53 Astrocytoma *IDH*-mutant. *TERT* promoter mutations were analyzed: in 124 GBM *IDH*-wildtype, 4 GBM *IDH*-mutant, 49 1p/19q codeleted gliomas, 41 Astrocytoma *IDH*-wildtype, 51 Astrocytoma *IDH*-mutant. *MGMT* methylation was analyzed: in 235 GBM *IDH*-wildtype, 10 GBM *IDH*-mutant, 49 1p/19q codeleted gliomas, 41 Astrocytoma *IDH*-wildtype and 54 Astrocytoma *IDH*-mutant

Following the reclassification of gliomas according to the 2016 WHO classification, we investigated the role of *TERT* promoter mutations, *EGFR* amplification, *PTEN* deletion and *MGMT* promoter methylation in molecular glioma subgroups. Here, we intended to assess whether these molecular alterations are predominantly altered in a specific subgroup, which could help to redefine the established molecular subgroups of gliomas. *EGFR* amplification was more frequently detected in *IDH-*wildtype gliomas, both, GBM (38%) and astrocytomas (43%), compared to *IDH*-mutant gliomas (11 and 4%, respectively). This molecular alteration was absent from the 1p/19q codeleted glioma subgroup (0%). *PTEN* deletions were identified in 83% of GBM *IDH*-wildtype, the most aggressive glioma group (Fig. 2), characterized by an OS of 10 months (Fig. 1). However, these alterations were also found in 43 and 50% of GBM *IDH*-mutant (OS:25 months) and astrocytomas *IDH*-wildtype (OS:14 months) respectively. In 1p/19q codeleted gliomas and astrocytomas *IDH*-mutant (OS:198 and 114 months, respectively), the two less aggressive subtypes of gliomas, the incidence of *PTEN* deletion was reduced (8 and 21% respectively). These results suggested that *PTEN* deletions are predominantly found in the most aggressive subgroups of gliomas. *TERT* promoter mutations were mainly found in 1p/19q codeleted (94%) and GBM *IDH-*wildtype (88%) molecular subgroups (Fig. 2), suggesting that this is not a good biomarker for diagnosis. Regarding *MGMT* promoter methylation status, we observed that: 100% of 1p/19q codeleted gliomas, 91% of astrocytomas *IDH*-mutant and 50% of GBM *IDH*-mutant samples were methylated. Therefore, *MGMT* promoter methylation, as expected, was inversely associated with aggressiveness, since it appears most frequently in groups with better prognosis (Fig. 2).

### Prognostic impact of *EGFR* amplification, *PTEN* deletion, *TERT* promoter mutations and *MGMT* promoter methylation

**Fig. 3:**
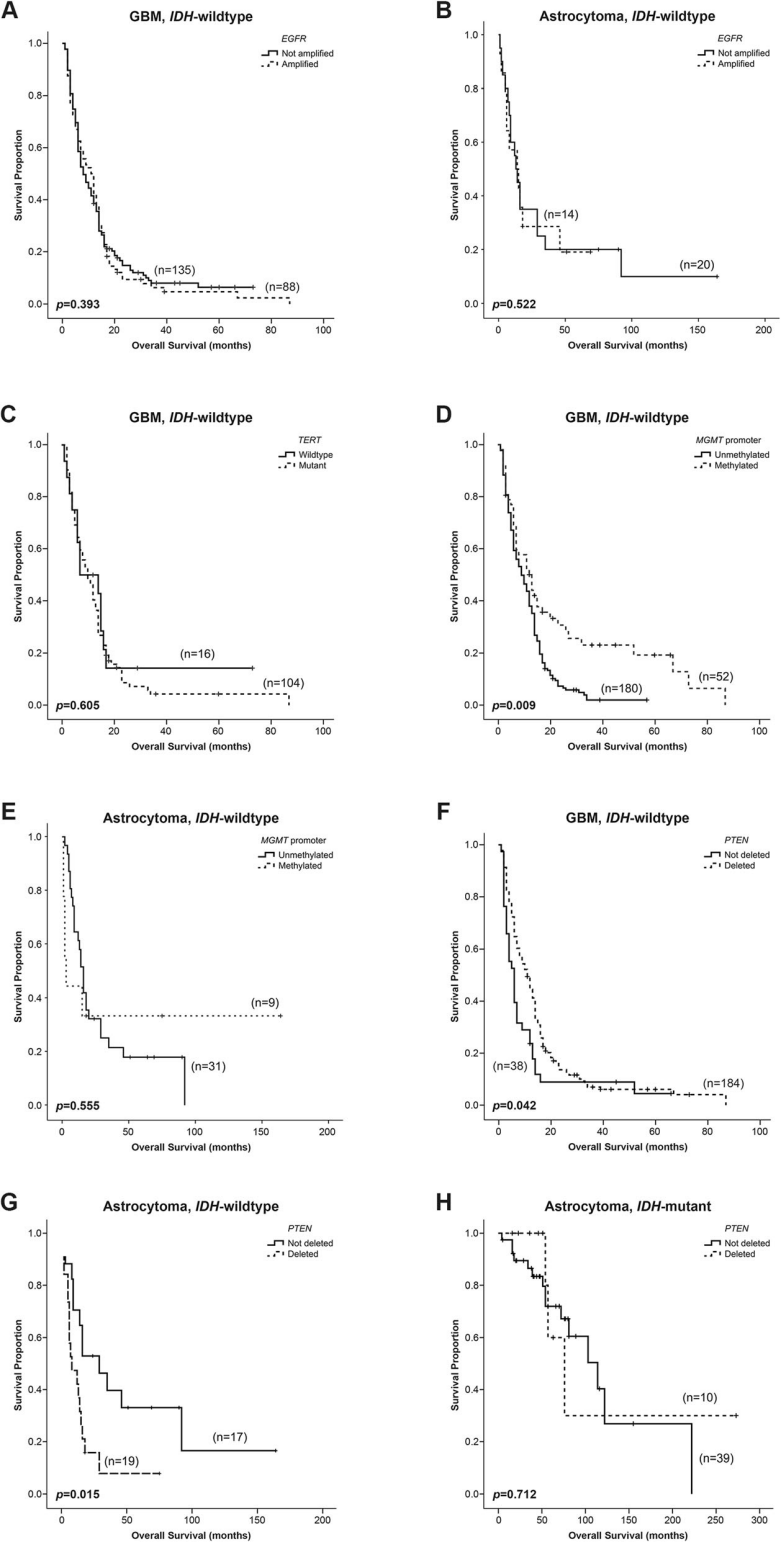
The impact of *EGFR* amplification, *TERT* promoter mutations, *PTEN* deletion and *MGMT* promoter methylation on overall survival of glioma patients. Kaplan-Meier curves of *EGFR* amplification in GBM *IDH-*wildtype (**a**) and in astrocytoma *IDH*-wildtype (**b**). Kaplan-Meier curves of *TERT* promoter mutations (**c**) and *MGMT* promoter methylation in GBM *IDH-*wildtype (**d**) and in astrocytoma *IDH*-wildtype (**e**). The impact of *PTEN* deletion in overall survival of GBM *IDH*-wildtype (**f**), astrocytomas *IDH-*wildtype (**g**) and *IDH*-mutant (**h**)

Furthermore, we evaluated the prognostic value of these distinct genetic alterations in each molecular subgroup of gliomas. We verified that *EGFR* amplification did not have significant impact in the overall survival of patients with GBM *IDH*-wildtype and astrocytoma *IDH*-wildtype, (*p* = 0.393 and *p* = 0.522, respectively) (Fig. 3a and b), as well as, *TERT* promoter mutations in GBM *IDH-*wildtype (*p* = 0.605), although the number of cases was too small for conclusive results (Fig. 3c).

However, as shown in Fig. 3d, *MGMT* promoter methylation was significantly associated with a prolonged overall survival in GBM *IDH*-wildtype (*p* = 0.009), while in astrocytomas *IDH*-wildtype (Fig. 3e) had no impact in prognosis (*p* = 0.555). The effect of this biomarker in astrocytomas *IDH*-wildtype could be related with the low number of methylated samples (*n* = 9).

Surprisingly, *PTEN* deletion had a dual effect in the prognosis of GBM and Astrocytomas *IDH*-wildtype. This molecular alteration was a favorable prognostic for GBM *IDH*-wildtype (*p* = 0.042) and a unfavourable prognostic for astrocytoma *IDH*-wildtype (*p* = 0.015) (Fig. 3f and g). Moreover, in astrocytomas *IDH*-mutant, *PTEN* deletion was not found to have a significant impact on overall survival (*p* = 0.702) (Fig. 3h). The multivariate analysis, considering *EGFR* amplification, *PTEN* deletion and *MGMT* methylation and controlling for age, gender and treatment, validated the role of *PTEN* deletion (Hazard Ratio (HR) =0.65; 95% CI 0.43–0.99) and *MGMT* promoter methylation (HR = 0.61; 95% CI 0.42–0.88) as independent factors of prognosis in GBM *IDH-*wildtype. In addition, also confirmed the role of *PTEN* deletion as a prognostic factor of poor outcome (HR = 4.48; 95% CI 1.34–14.94) (Table 4).

### The predictive effect of *EGFR* amplification, *PTEN* deletion and *MGMT* promoter methylation in GBM *IDH*-wildtype patients

**Fig. 4:**
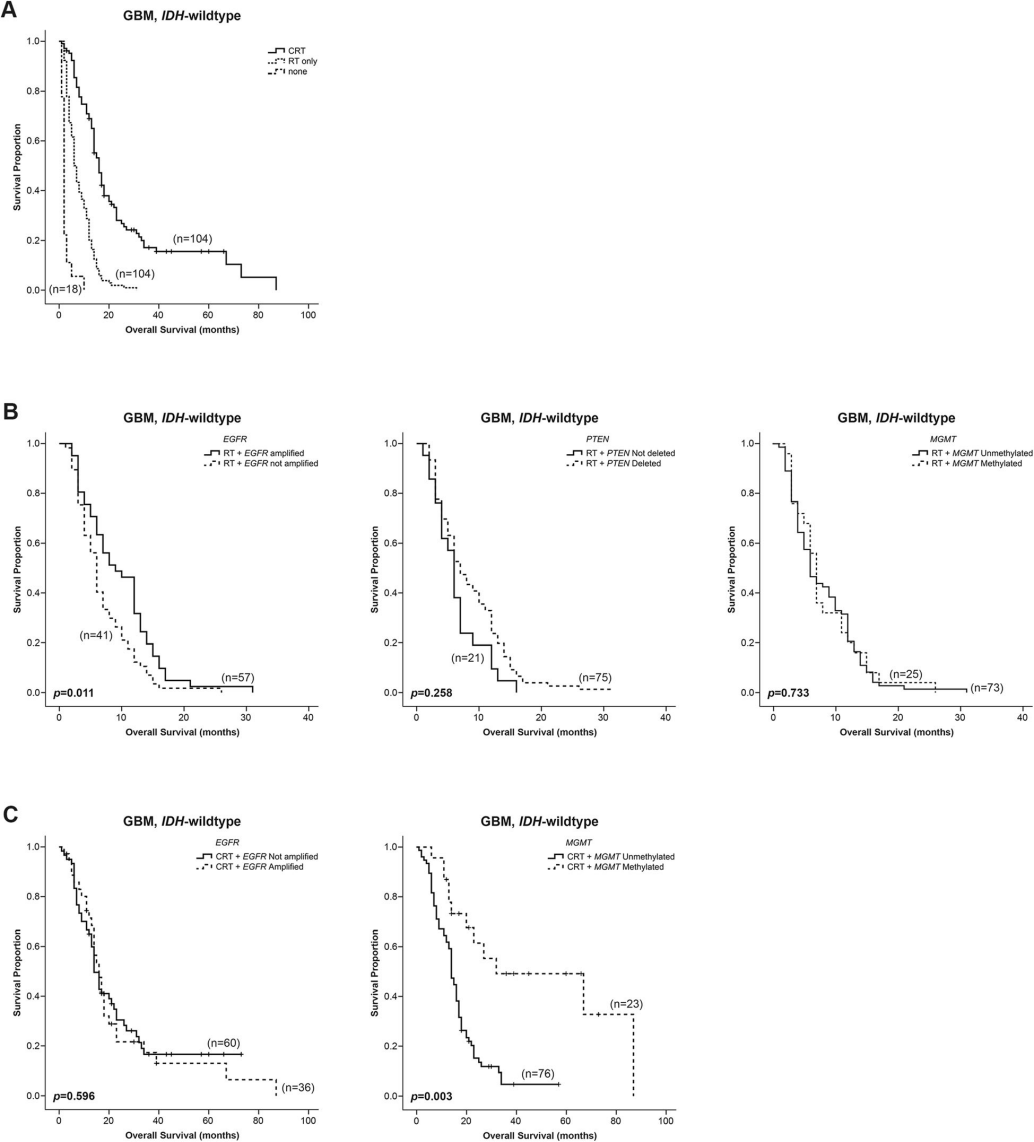
Kaplan – Meier survival estimates of overall survival according to the *EGFR* amplification, *PTEN* deletion and *MGMT* methylation status and random assignment to Chemoradiotheraphy (CRT) or Radiotherapy (RT) in patients with *GBM IDH-wildtype*. The survival curves of glioma’s patients according to treatment received (**a**). The impact of *EGFR* amplification, *PTEN* deletion and *MGMT* methylation in radiotherapy response using GBM *IDH-*wildtype subgroup (**b**). The impact of *EGFR* amplification and *MGMT* methylation in chemo-radiotherapy response using GBM *IDH-*wildtype subgroup (**c**)

To gain further insight into the predictive value of these biomarkers in molecular subgroups of gliomas, we analyzed the effect of *EGFR* amplification, *PTEN* deletion and *MGMT* promoter methylation in the response to therapy using the only group with representative samples - GBM *IDH*-wildtype (Fig. 4). However, in this group due to the small number of *TERT* wildtype samples, it was not possible to do this analysis regardless *TERT* promoter mutations.

Initially, we analyzed the OS of each group of patients treated with radiotherapy (RT) or chemo-radiotherapy (CRT), which was 6 and 16 months, respectively (Fig. 4a). CRT based on temozolomide is the standard treatment for patients with GBM. Patients subjected to RT alone, usually respect the following criteria: age above 70 years, other pathological conditions contra-indicating chemotherapy or a more severe clinical presentation. Patients who were not treated with RT or CRT, were directly to palliative care.

In patients with GBM *IDH*-wildtype, surprisingly the *EGFR* amplification was associated with a better response to radiotherapy (*p* = 0.011) (Fig. 4b left), however it was unable to predict the response to chemo-radiotherapy (*p* = 0.596) (Fig. 4c right). The multivariate analysis performed in RT subgroup, considering the three genetic alterations and controlling for age and gender revealed that *EGFR* amplification constitutes an independent predictive factor of response to radiotherapy (HR = 0.56; 95% CI 0.36–0.88) (Table 5). This result suggests a new putative strategy for the management of patients, who may have a better response to radiotherapy, although it should be validated in other cohorts.

**Table 5:** Multivariate analysis for the predictive value of *PTEN* deletion, *MGMT* methylation and *EGFR* amplification in GBM *IDH* wildtype

	Median Survival (MS-months)	N	Multivariate analysis^a^		
			Hazard ratio	95% Cl	*p*-Value
*GBM IDH-wildtype*					
CRT	16.0	104	0.053	0.029–0.098	< 0.001
RT	6.0	104	0.181	0.105–0.314	< 0.001
None	2.0	18		Reference	
RT					
*PTEN*					
Deleted	7.0	74	0.74	0.45–1.24	0.258
Non-deleted	6.0	20		Reference	
*MGMT*					
Methylated	7.0	24	1.09	0.67–1.78	0.733
Unmethylated	6.0	70		Reference	
*EGFR*					
Amplified	9.0	39	0.56	0.36–0.88	0.011
Non-amplified	6.0	55		Reference	
*CRT*					
*PTEN*					
Deleted	14.0	84	1.68	0.40–7.15	0.481
Non-deleted	14.0	5		Reference	
*MGMT*					
Methylated	32.0	20	0.34	0.17–0.69	0.003
Unmethylated	14.0	69		Reference	
*EGFR*					
Amplified	15.0	32	0.88	0.54–1.43	0.596
Non-amplified	14.0	57		Reference	

^a^Multivariate analysis was performed controlling the following independent variables: age and gender. *RT* Radiotherapy, *CRT* Chemo-radiotherapy

The effect of *PTEN* deletion on response to therapy was inferred only in patients exposed to radiotherapy, since most patients submitted to chemo-radiotherapy had *PTEN* deleted. According to our results, *PTEN* deletion had no predictive value in the response to radiotherapy (*p* = 0.258) (Fig. 4b middle).

Additionally, our results showed that *MGMT* methylated samples were associated with an improved response to chemo-radiotherapy compared to *MGMT* unmethylated samples in GBM *IDH-*wildtype patients (*p* = 0.003) (Fig. 4c right). *MGMT* methylation constitutes a well-known predictive biomarker of gliomas used to infer which patients would have a better response to chemotherapy with temozolomide. Despite of its effects in response to chemo-radiotherapy, as described before [25], *MGMT* was not an important predictor of response to radiotherapy alone (*p* = 0.733) (Fig. 4b right).

## Discussion

In the present study we evaluated the impact of the new 2016 WHO classification of Central Nervous System Tumors in a 444 diffuse gliomas cohort, previously classified according to the 2007 WHO classification based on histological features.

Our results showed a decrease in the percentage of oligodendrogliomas, from 18.5% of the samples previously diagnosed using the histological classification, to 11% of the samples according to the new classification. On the other hand, there was an increase in the percentage of astrocytomas (from 11 to 22.1% of the samples). This main alteration in glioma subgroups was associated with the introduction of 1p/19q codeletion and *IDH* status, which were decisive in the subdivision of astrocytoma and oligodendroglioma as well as the disintegration of the oligoastrocytoma group. These results are in accordance with the study of Iuchi et al., which reported astrocytoma and oligodendroglioma subgroups as the main targets of the 2016 WHO classification effect [32]. However, according to Tabouret and co-authors, the reclassification of the French cohort showed a similar frequency of oligodendrogliomas before and after the reclassification of gliomas (31.6–34.5%, respectively), while the number of GBM (33.8–50.3%) and astrocytomas (7–16.2%) increased [33]. The differences observed between our study and the French cohort, could be related with the reclassification of oligoastrocytomas, since in our study most oligoastrocytomas were reclassified as astrocytoma (*n* = 35), while in the study of Tabouret et al. the vast majority of oligoastrocytomas were considered GBM [33].

Even with the introduction of molecular biomarkers, the distribution of patients previously diagnosed with oligoastrocytomas remains a difficult task, which is demonstrated by the variability between studies [32, 33]. In our study 10 samples of oligoastrocytomas were included into the NOS subgroup. The analysis of alpha thalassemia/mental retardation syndrome X-linked (*ATRX*) loss and tumor protein 53 (*TP53)* mutations was not performed, which constitutes a limitation of this study. The analysis of these both biomarkers is suggested in the 2016 WHO classification only in doubtful cases [4], and they are currently done in our Institute by immunochemistry. Actually, the mutational status of these genes is not determined in diagnosis of gliomas for two main reasons: i) by itself are unable to identify the subtype of glioma sample and because; ii) it is expensive, they constitute long genes, becoming difficult their analysis using the conventional molecular techniques.

In total, 41 samples of our cohort were inserted into the NOS glioma subgroup, highlighting the need for new biomarkers, in order to be possible to classify gliomas with 1p or 19q deletion and gliomas *IDH*-wildtype with 1p/19q codeletion. Although, it is important to note the most of them are included in this subgroup due to technical issues.

Here, as expected, GBM constituted the most prevalent type of glioma (57.7%), like previously reported by Iuchi et al. (66%), Tabouret et al. (50%) and Ostrom et al. (45%) [1, 32, 33]. However, GBM *IDH-*mutant accounted for only 2.5% of all GBM, slightly less than the 10, 17.2 and 7.8% previously reported [32–34]. In addition, astrocytomas *IDH-*wildtype and *IDH*-mutant showed similar frequencies (9.7 and 12.4% respectively), slightly different from the results reported by Iuchi and co-authors (13.7% of astrocytomas *IDH-*wildtype and 6.7% of astrocytomas *IDH-*mutant) and Tabouret et al., (11% astrocytomas *IDH-*mutant and 5.3% of astrocytoma *IDH-*wildtype) [32, 33]. The differences detected in astrocytomas diagnosis could be related to their reclassification in GBM, which presently depends only on the histological features of the tumor, introducing some variability between studies.

Notably, 1p/19q codeleted gliomas were the molecular subgroup associated with the longest overall survival (OS:198 months), regardless of whether they were classified as oligodendrogliomas grade II (OS:172 months) or grade III (OS:97 months). These results suggested that 1p/19q codeletion is a strong biomarker of prognosis and even better than histological classification, since could embrace less aggressive tumors. In addition, these statements are in accordance with previous studies indicating 1p/19q codeletion was associated with a better prognosis when compared to non-codeleted tumors [35, 36].

Furthermore, *IDH* mutational analysis divided the astrocytoma group into two subgroups with distinct prognoses (*p* < 0.001), as previously reported [4, 37]. Here, *IDH* mutations did not had prognostic impact in GBM (*p* = 0.092) (Table 5), which could be explained by the reduced number of GBM *IDH-*mutant samples (*n* = 11).

We also evaluated the impact of *EGFR* amplification, *PTEN* deletion, *TERT* promoter mutations and *MGMT* promoter methylation in the diagnosis, prognosis and response to therapy of patients with diffuse gliomas. As reported by other studies, *EGFR* amplification was more common in *IDH*-wildtype gliomas [38–40]. To date, most studies evaluated the prognostic value of *EGFR* amplification using only the histological diagnosis, instead of the molecular subgroups of gliomas [21–23]. In this work, we reported for the first-time that *EGFR* amplification had no significant prognostic value in molecular subgroups of gliomas - *IDH*-wildtype GBM and astrocytomas. Nevertheless, we only evaluated the presence of *EGFR* amplification and not *EGFR* activating mutations. Previously, it was described that tumors with both *EGFRvIII* overexpression and *EGFR* amplification constitute an indicator of poor prognosis in GBM patients [23]. The prognostic value of *EGFR* was also associated with patient’s age, seeming to be correlated with worse outcomes in younger patients [41]. However, none of these studies considered the mutational status of *IDH*, which means that the effect on prognosis by *EGFR* amplification may be dependent of *IDH* mutations.

Additionally*, EGFR* amplification has been appointed as one of the causes for the development of radio-resistance in gliomas [42]. Most interestingly, we found that patients with GBM *IDH*-wildtype and *EGFR* amplification had a significantly better overall survival than those without *EGFR* amplification, only when treated with radiotherapy alone, and not when treated with chemo-radiotherapy. At this point, the clinical significance of this finding, and the reasons why it did not occur with chemo-radiotherapy, are not fully understood. However, this result should be validated in other cohorts with a higher number of samples. Nevertheless, our observation is consistent with the previously described in non-small cell lung cancer, where the presence of *EGFR* activating mutations and also *EGFR* amplification were associated with a radiosensitive phenotype, inducing increased levels of pro-apoptotic proteins and reduced capability to repair DNA [43–45].

The relative frequency and prognostic value of *PTEN* deletion in diffuse gliomas were analyzed using histological diagnosis, which explains the variability between the reported studies [16, 17]. Interestingly, the observed role of *PTEN* deletion in prognosis of GBM and astrocytomas *IDH*-wildtype has never been documented. In our study, *PTEN* deletion was considered a factor of good prognosis in GBM *IDH*-wildtype (*p* = 0.042), although using a reduced number of samples without *PTEN* deletion (*n* = 38 vs *n* = 184 with *PTEN* deleted). Despite this, it was previously noticed that *PTEN* loss could be associated with a more favorable prognosis, since it leads to a better response to chemotherapy by compromising homologous recombination of DNA, through the transcriptional regulation of Rad51 [16, 46]. Another hypothesis to the observed result in our study may be the absence of an inverse correlation between *PTEN* expression and AKT activity, as demonstrated in melanoma and breast cancer [47, 48]. Moreover, this dual effect of *PTEN* deletion in prognosis could be related with the specific tyrosine which is the target of *PTEN* phosphorylation [49]. This hypothesis would explain why *PTEN* deletion predicts a good outcome in GBM *IDH*-wildtype. In contrast, in astrocytomas *IDH*-wildtype the deletion of *PTEN* is a factor of poor prognosis, as expected, since this is a tumor suppressor gene. Further work should be undertaken to evaluate the mechanisms through which this molecular alteration differentially affects the prognosis of these both groups of gliomas.

In this dataset, *TERT* promoter mutations did not have prognostic value in GBM *IDH-*wildtype, which is consistent with Nguyen et al. and Eckel-Passow et al. previous studies [17, 18]. Eckel-Passow et al. reported that *TERT* promoter mutations are associated with a poor prognosis in the absence of *IDH* mutations in grade II and III gliomas [18]. Therefore, this study verified that *TERT* promoter mutations would be an important biomarker in grade II and III gliomas. However, these results are not according to the 2016 WHO classification. The authors were not considering a subgroup of 1p/19q codeletion and *IDH* mutation, as an independent group [18]. Interestingly in our results 1p/19q codeleted gliomas showed a higher percentage of *TERT* promoter mutations – 94%. Therefore, this suggests that the effect observed on overall survival by Eckel-Passow et al., could be associated with the difference between 1p/19q codeleted/*IDH* mutant gliomas and astrocytomas independently of *TERT* promoter mutations.

*MGMT* promoter methylation is a biomarker extensively studied in GBM. As previously mentioned, this biomarker is a prognostic factor of prolonged overall survival [24, 26]. Here, *MGMT* methylation was found mainly in *IDH*-mutant gliomas, which is in accordance with the literature [3, 27]. *IDH* mutations are responsible for increased levels of 2-hydroxiglutarate, which in turn determines the inhibition of several enzymes, such as Jumonji-C domain-containing histone lysine demethylases [50]. It is already known that *MGMT* methylation is associated with better outcomes in both *IDH* mutant and *IDH* wildtype GBM [27, 28], although it only constitutes a predictive biomarker for the benefit to temozolomide chemotherapy and not to radiotherapy in patients with GBM-*IDH* wildtype [25]. Interestingly, Rivera et al., reported the predictive value of *MGMT* methylation to radiotherapy response in GBM patients, independently of the *IDH* mutational status [51].

## Conclusions

In this work we demonstrated that the 2016 WHO classification brought an improvement in the accuracy of diagnosis and prognosis of diffuse gliomas, validating the importance of adding molecular characteristics to histology. However, this new classification has limitations to stratify these heterogeneous tumors, for instance, GBM *IDH*-wildtype subgroup had a higher disparity in patients’ survival as well as astrocytomas *IDH* wildtype. Another important issue is gliomas NOS, which embrace gliomas with 1p/19q codeleted but *IDH* wildtype or even tumors with partial deletion (1p or 19q). Furthermore, this study highlighted the clinical importance of gathering additional biomarkers in the diagnostic routine of gliomas. *TERT* promoter mutations seem not to confer additional information about gliomas diagnosis and prognosis. In GBM *IDH*-wildtype molecular subgroup, *PTEN* deletion seems to be important for prognosis and *EGFR* amplification for radiotherapy response. In astrocytoma *IDH*-wildtype, *PTEN* deletion appear to be important in prognosis. These interesting findings should be validated in other cohorts as well as the in vitro studies should be performed to clarify the molecular mechanisms behind this biological behavior. In contrast, *MGMT* promoter methylation has been shown to be a strong biomarker of prognosis and a predictor of response to chemotherapy, reinforcing the idea that this biomarker should be include in glioma classification, resulting in a new molecular subgroup within the GBM *IDH*-wildtype.

## Data Availability

The datasets analyzed during the current study are available from the corresponding author on reasonable request.

## Abbreviations

ATRX: Alpha thalassemia/mental retardation syndrome X-linked
CRT: Chemo-radiotherapy
EGFR: Epidermal Growth Factor Receptor
FISH: Fluorescent In Situ Hybridization
GBM: Glioblastomas
HR: Hazard Ratio
IDH: Isocitrate dehydrogenase
IPOLFG: Unidade de Investigação em Patobiologia Molecular of Instituto Português de Oncologia de Lisboa Francisco Gentil
ISCN: International System of Human Cytogenetic Nomenclature
MGMT: O-6-methylguanine-DNA methyltransferase
MLPA: Multiplex Ligation-dependent Probe Amplification
NOS: Not Otherwise Specified
PCR: Polymerase Chain Reaction
PI3K-Akt: Phosphatidylinositol-3-kinase -protein kinase B
PTEN: Phosphatase and Tensin homologue
RT: Radiotherapy
TERT: Telomerase Reverse Transcriptase
TP53: Tumor Protein 53
WHO: World Health Organization
